# The Impact of Levothyroxine and Testosterone Administration on Bladder Contractility in the Rat Model of Propylthiouracil-Induced Hypothyroidism

**DOI:** 10.5152/tud.2025.24099

**Published:** 2025-01-03

**Authors:** Didem Yılmaz Oral, Berna Güven Ciloğlu, Serap Gür

**Affiliations:** 1 Department of Pharmacology, Çukurova University, Faculty of Pharmacy, Adana, Türkiye; 2 Department of Pharmacology, Ankara University, Faculty of Pharmacy, Ankara, Türkiye

**Keywords:** Hypothyroidism, levothyroxine, testosterone, urinary bladder

## Abstract

**Objective:**

To investigate the effects of testosterone (T) treatment, with or without levothyroxine, the most widely used and least effective medication for managing hypothyroidism, on the functional and histological changes in propylthiouracil (PTU)-induced hypothyroid rat bladders.

**Methods:**

Male rats (n = 35) were split into control, hypothyroid, hypothyroid rats treated with levothyroxine (20 µg/kg/day, oral, 2-weeks), hypothyroid rats treated with Sustanon (10 mg/kg,iIM, once/week, 2-weeks), and hypothyroid rats treated with combined treatment groups. Hypothyroidism was induced by PTU (0.05% in drinking water, 6 weeks). The serum concentration of triiodothyronine (T3), thyroxine (T4), total T, and the detrusor muscle’s contractile responses were determined. A morphological study was conducted using Masson trichrome staining.

**Results:**

Triiodothyronine, T4, and T levels were considerably reduced in rats with hypothyroidism; however, these hormones were restored by levothyroxine and Sustanon. Compared to controls, the combination therapy improved the ratio of smooth muscle to collagen and the contractile responses to carbachol, electrical field stimulation, and adenosine triphosphate in the hypothyroid bladder.

**Conclusion:**

The authors’ research suggests that hypothyroidism may affect the contractility and morphology of the bladder. In males with hypothyroidism and urogenital system dysfunction, combination therapy with thyroid hormones and T has a major impact on repairing detrusor smooth muscle contractility and bladder histomorphology.

**Main Points**

- Hypothyroidism and low T levels are likely to impair bladder contractility.
- The combined treatment with levothyroxine and Sustanon improved hypothyroidism-induced bladder dysfunction.
- Levothyroxine and Sustanon treatment have a synergistic effect on bladder structural modification.

## Introduction

Thyroid and thyroid-stimulating hormones (TSHs) are necessary for smooth muscles, blood vessels, glomerular filtration, and urine function.^1^ Earlier data showed a connection between thyroid disease and abnormalities of the kidneys and urinary system.^2^ Urinary dysfunctions, including difficulties such as micturition and urine retention, have been connected to hypothyroidism.^3,4^ The hypothyroidism-related neurological and muscular dysfunction may have an impact on the synchronized activation of pelvic floor muscle reflexes during micturition.^4^ Furthermore, there have been reports of paralytic ileus occurring with bladder atony in individuals with hypothyroidism.^5,6^ In hypothyroid rabbits, somatovisceral reflexes that coordinate micturition and voiding efficiency were also impaired.^4^ Moreover, earlier data stated that there was bladder hypotonia in patients with hypothyroidism, and bladder tonus was regulated after thyroid function was normalized.^3^ Restoration of normal thyroid function increased the frequency of micturition in individuals with hypothyroidism.^4^ Similarly, hypotonia and low urinary bladder are restored with levothyroxine treatment.^3,4^

Hypothyroidism is likely to be linked to low serum testosterone (T) levels.^7^ Moreover, low T levels are linked to lower urinary tract symptoms (LUTSs), and the connection between T deficiency and bladder dysfunction has not been established.^8^ Testosterone replacement therapy (TRT) has been shown to have a positive impact on bladder function and the reversal of wall changes in individuals with late-onset hypogonadism and urogenital dysfunction.^9^ This is achieved by increasing urinary bladder smooth muscle.^10^

Only a few studies have examined how thyroid hormones and T affect bladder function. This research aimed to examine the impacts of levothyroxine, a commonly used and effective medicine for treating hypothyroidism, with or without T treatment, on the functional and histological changes in the rat bladder in hypothyroidism induced by propylthiouracil (PTU).

## Material and Methods

### Animals

Male Wistar rats (n = 35, 10-week, weighing 387.0 ± 74.7 g) were purchased from the Ankara University School of Medicine Experimental Animal Breeding and Research Laboratory in Ankara, Türkiye. They were then split into 5 groups: (a) control rats; (b) hypothyroid rats; (c) hypothyroid rats treated with levothyroxine; (d) hypothyroid rats treated with Sustanon (4 types of T combination: propionate, phenylpropionate, isocaproate, and decanoate); and (e) hypothyroid rats treated with levothyroxine and Sustanon. The rats were administered PTU in their drinking water (0.05%) for 6 weeks to induce a hypothyroid model. Four weeks following PTU administration, levothyroxine (20 µg/kg/day, oral) and Sustanon (10 mg/kg IM, once/week) were administered for 2 weeks.^11^ The rats were individually kept in separate cages and given unrestricted access to food and drink in a room with a regulated temperature (22 ± 1°C) artificially lit from 7:00 am to 7:00 pm everyday. Ankara University Ethics Committee approved the experimental processes involving the rats (Approval no.: 2023-18-166; Date: 2023).

### Isometric Tension Measurements

The animals were killed under anesthesia (ketamine/xylazine; 100/10 mg/kg, intraperitoneally). The bladder was separated from the posterior face by cutting it into strips that were 1 cm long and 2 mm wide. The isolated bladders were placed in an organ bath containing Krebs solution and 95% O_2_ / 5% CO_2_ at 37°C while under resting tension (1 g). A metal hook and a force transducer were connected to the strips. Using 2 platinum electrodes (Grass Instruments, Quincy, MA, USA), an electrical pulse (5 ms pulse width, amplitude 90 V) was administered for 15 seconds at increasing frequencies (1-40 Hz) for electrical field stimulation (EFS). The bladder strips were contracted with KCl (60 mmol), cumulative carbachol (10^−7^-10^−4^ M), EFS (1-40 Hz), and adenosine triphosphate (ATP) (0.1-1 mM) after an hour of equilibration. The contractile response was standardized to a percentage of this value.^12^

### Total Testosterone, Triiodothyronine, and Thyroxine in Rat Plasma Measurements

After the rats were sacrificed, blood samples were taken from each group to measure the levels of total T, Triiodothyronine (T3), and Thyroxine (T4). Using a Multi-Crystal Gamma Counter (LB 211) (Berthold, Germany), the radioimmunoassay kit (RIA, Beckman Coulter kit) was used to analyze total T (ng/mL). The blood was immediately centrifuged for 20 minutes at 3000 rpm, and the obtained plasma was kept cold until analysis. The UniCel DxI 800 Access Immunoassay System (Beckman Coulter Inc., Brea, CA, USA) was used to assess the levels of T3 and T4 (ng/dL).

### Masson’s Trichrome Staining

Bladder tissues embedded in paraffin (4-6 μm) were stained using Masson’s trichrome kit (Sigma Chemical, St. Louis, MO) and photographed using a color digital camera system and a light microscope (Leica Microsystems, Wetzlar, Germany). Computerized imaging software (Image J, NIH, Bethesda, MD) was used to examine the pictures for 2 different populations of smooth muscle fibers and collagen.

### Statistical Analysis

The data values were analyzed using Prism v.4 software (GraphPad Software, San Diego, CA, USA) and reported as the mean ± SEM. The results obtained from several groups were compared using a one-way analysis of variance (ANOVA) followed by Bonferroni analysis. The minimum level of significance was set at *P* < .05.

## Results

### Characteristics of Animals

All hypothyroid rats had lower body weights than control rats (Table 1). Both the administration of monotherapy and combination treatment failed to restore body weight levels in rats with hypothyroidism. No significant variation in bladder weight was seen among the groups (Table 1).

### Total Testosterone, Triiodothyronine, and Thyroxine Levels

The hypothyroid group had declined all serum hormone levels (total T, T3, and T4) compared to controls (*P* < .001, Table 1). The administration of levothyroxine as a single therapy increased T3 and T4 levels (*P* < .01 and *P* < .001 vs. PTU-treated rats). Sustanon monotherapy raised the total T level (*P* < .001 vs. PTU-treated rats). The combination of levothyroxine and Sustanon treatment led to a substantial elevation in all hormone levels (Table 1).

### The Urinary Bladder Contractile Responses

**Figure 1.**
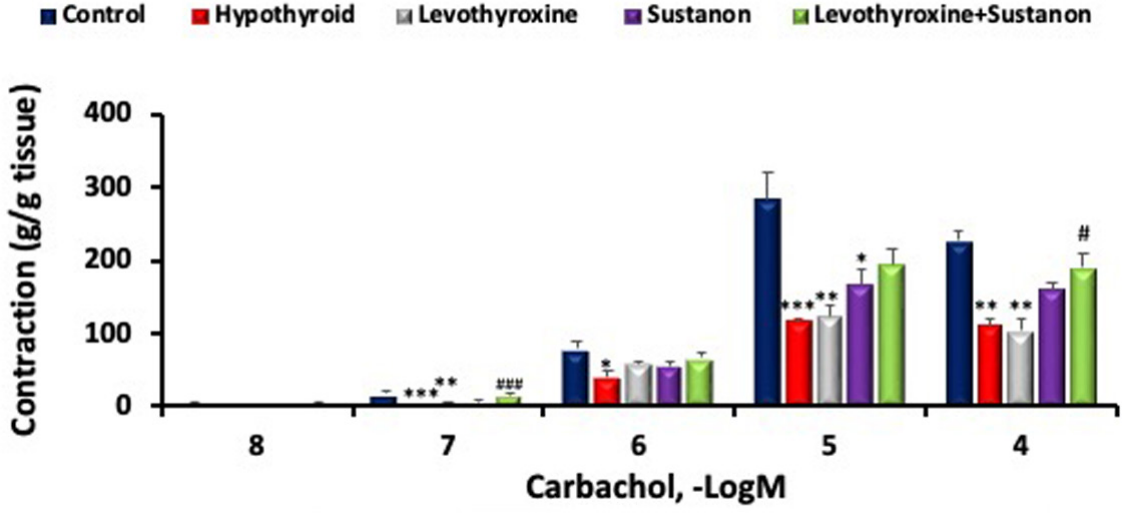
Contractile dose–response curves for carbachol in bladder strips from all groups. Data are presented as mean ± SEM (n = 7 rats) per group. *P < .05, **P < .01, ***P < .001 vs. the control group; ^#^*P* < .05, ^###^*P* < .001 vs. the hypothyroid group (ANOVA with Bonferroni post hoc test).

Carbachol-induced contractile responses (0.1, 1, 10, and 100 µM) in hypothyroid bladder strips were diminished compared to the control group (maximum response: 48%, *P* < .01 vs. controls, Figure 1). In addition, contractile responses to carbachol in levothyroxine-treated hypothyroid rats were reduced by 55% at 100 µM compared to controls (*P* < .01, Figure 1). The hypothyroid rats that received Sustanon treatment exhibited reduced contractile responses generated by carbachol compared to controls. Nevertheless, except at 10 µM, the difference was not statistically significant (*P* < .05 vs. controls, Figure 1). The combined medication of levothyroxine + Sustanon enhanced the decreased contractile response to carbachol (Figure 1).

**Figure 2.**
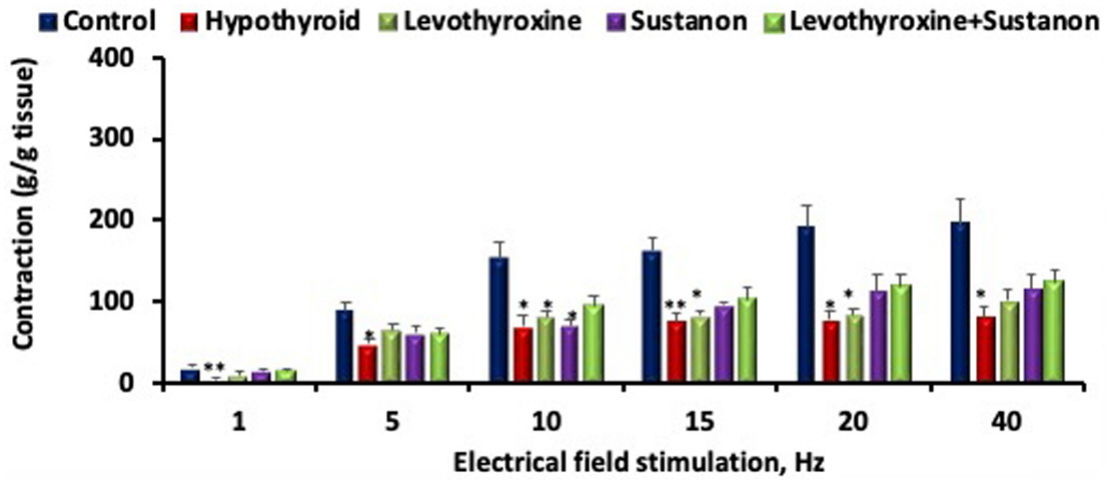
Contractile dose–response curves for EFS in bladder strips from all groups. Data are presented as mean ± SEM (n = 7 rats) per group. *P < .05, **P < .01 vs. the control group (ANOVA with Bonferroni post hoc test).

The presence of hypothyroidism resulted in a decrease in the contractile response to EFS in bladder strips (*P* < .05 vs. controls, Figure 2). Levothyroxine treatment for hypothyroid rats resulted in a significant decrease in the contraction responses elicited by EFS at frequencies of 10, 15, and 20 Hz compared to control rats. Furthermore, Sustanon therapy significantly enhanced EFS-induced contraction at all voltage levels except the 10 Hz frequency. Moreover, the normalization of the decrease was achieved with the implementation of combination therapy (Figure 2).

**Figure 3.**
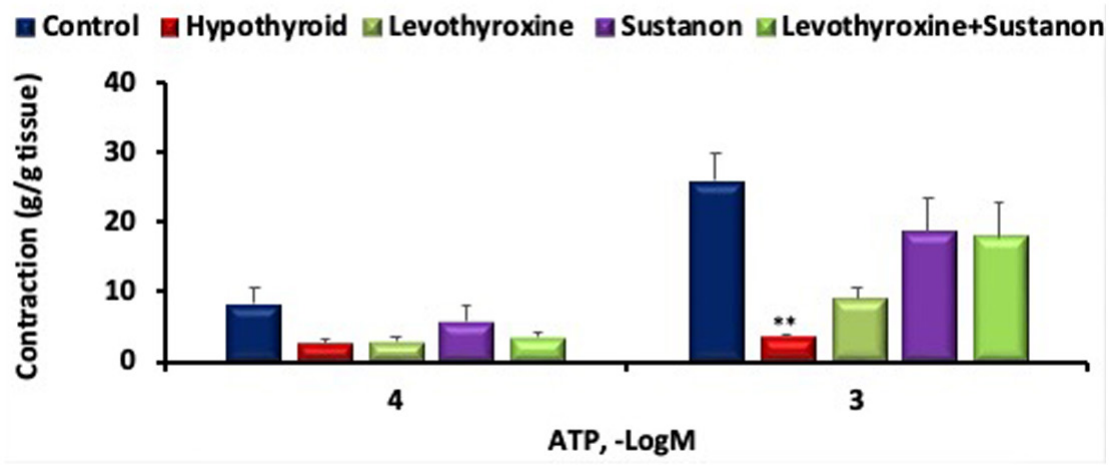
Contractile dose–response curves for ATP in bladder strips from all groups. Data are presented as mean ± SEM (n = 7 rats) per group. **P < .01 vs. the control group (ANOVA with Bonferroni post hoc test).

Hypothyroidism caused a significant decrease in the purinergic agonist ATP-induced contractile response at 1 mM (*P* < .01 vs. controls, Figure 3). The ATP-induced contractile response at a 1 mM dosage in the hypothyroid group receiving levothyroxine alone was non-significantly lower than in controls (Figure 3). Contractile responses to ATP did not vary between the treatment groups (Figure 3).

**Figure 4.**
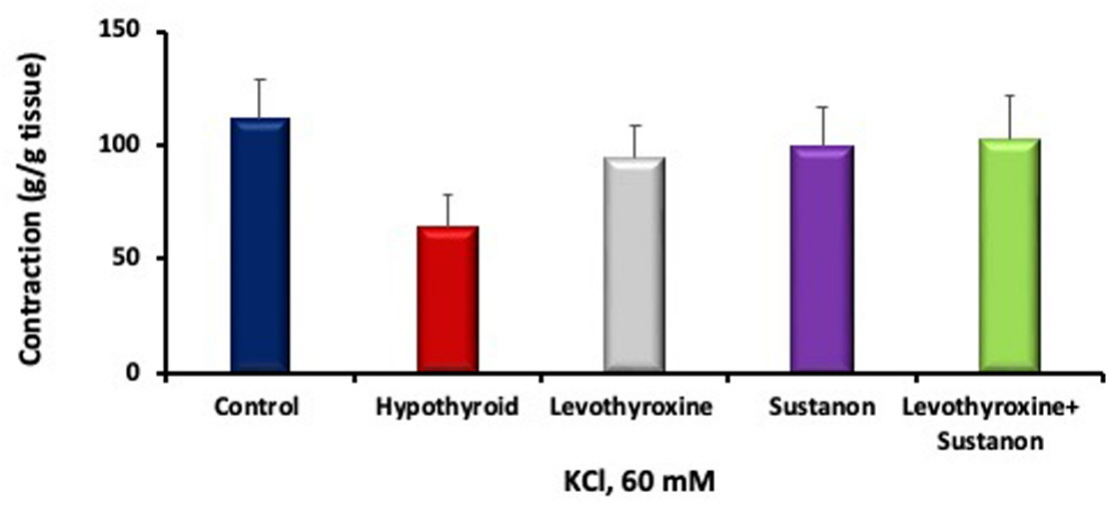
Contractile dose–response curves for KCl in bladder strips from all groups. Data are presented as mean ± SEM (n = 7 rats) per group (ANOVA with Bonferroni post hoc test).

The hypothyroid group exhibited reduced contraction levels caused by the membrane depolarizing agent KCl in comparison to both the control group and all treatment groups. However, this decrease did not achieve statistical significance (Figure 4).

### Smooth Muscle Cells/Collagen Ratio

**Figure 5.**
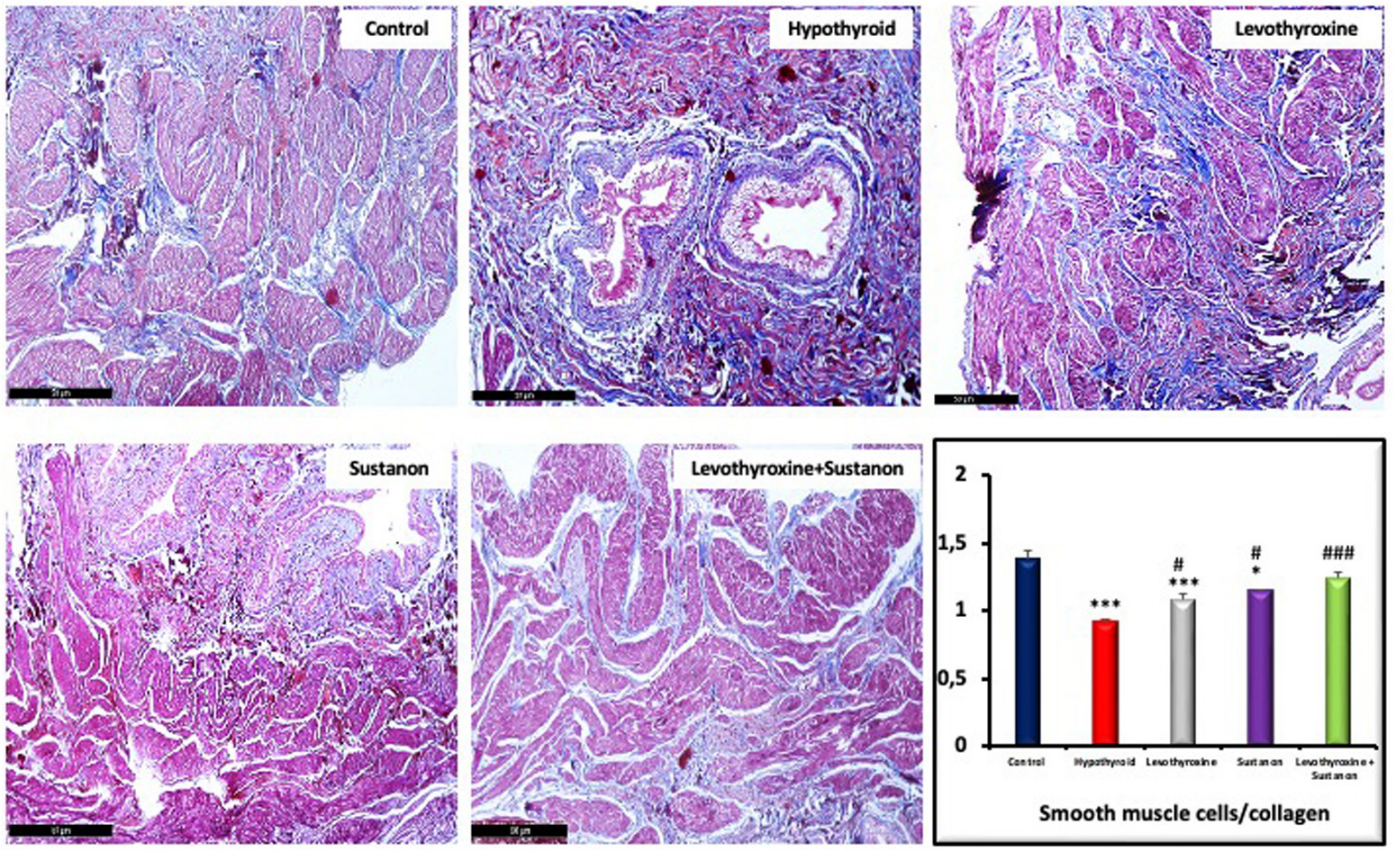
Masson’s trichrome staining of bladder tissues from all groups. Smooth muscle and connective tissues are stained in red and blue, respectively. The ratio of smooth muscle cells (SMC)/collagen was displayed in the bar graph. Data are the mean  ±  SEM (n  =  4 rats). *P < .05, ***P < .001 compared with the control group; ^#^*P* < .05, and ^###^*P* < .001 vs. the hypothyroid group (ANOVA with Bonferroni post hoc test).

The smooth muscle cell-to-collagen ratio in hypothyroid and levothyroxine-treated rats was reduced when compared with control rats (*P* < .001; Figure 5). Sustanon therapy alone partly reversed the decline (*P* < .05 vs. hypothyroid; Figure 5). Furthermore, a complete restoration was achieved with levothyroxine and Sustanon treatment (*P* < .001 vs. hypothyroid, Figure 5).

## Discussion

The consequences of low T levels and hypothyroidism on bladder functioning are being shown for the first time in this investigation. The present study revealed significant findings as follows: the PTU-induced hypothyroidism model showed decreased T3, T4, and T levels, while combination treatment with levothyroxine and Sustanon increased all hormone levels. In the context of tension recordings, the utilization of combination therapy led to an enhancement of the diminished contractile response to carbachol, EFS, and ATP in bladder strips affected by hypothyroidism. Additionally, comparing the bladders of hypothyroid and levothyroxine-treated rats to those of control and levothyroxine- and Sustanon-treated rats, the ratio of smooth muscle to collagen was lower.

The reduction in the body weight of all hypothyroid groups is similar to previous data.^11,13^ Furthermore, PTU administration declined T, T3, and T4 levels in rats, matching the results of earlier studies.^14,15^ According to the results, T levels were not normalized after levothyroxine treatment. In men with hypothyroidism, thyroid hormone therapy might not be sufficient to restore normal T levels; instead, TRT could be required.

In the current study, PTU-induced hypothyroidism reduced contraction responses to carbachol, an M2 muscarinic receptor agonist, in the bladder tissue. Furthermore, levothyroxine treatment did not return the contractile response to carbachol, and the combination treatment was more effective in improving the carbachol-induced contractile response in PTU-induced hypothyroidism than either drug alone. A previous study showed that thyroxine administration increased the decrement in muscarinic receptor agonist, acetylcholine-caused contraction in the thyroidectomized rat bladder.^16^ Also, the castrated rat bladder displayed decreased carbachol-induced contractile responses, and T treatment normalized the decrement.^17^ These findings suggest that hypothyroidism is likely to reduce muscarinic receptor-mediated contractility in smooth muscle, and the M2 muscarinic receptor agonist contractile responses are more associated with serum T levels than the regulation of T4 levels.

In the current investigation, PTU-induced hypothyroid rats showed a reduction in EFS-mediated contraction. Previous research showed that control and thyroidectomized rats did not differ in the EFS-induced contractile response in the bladder strips.^18^ Furthermore, the contractile response in the isolated corpus cavernosum obtained from the rats treated with PTU exhibited a significant increase when compared to the control group.^11^ The discrepancy may be elucidated by the experimental model of hypothyroidism and its impact on various tissues. Furthermore, combination treatment more effectively enhanced the decreased EFS contractions compared to single treatment regimens. However, no previous studies evaluated the effect of hormone replacement therapy on hypothyroidism-induced bladder dysfunction. Based on the findings, it can be concluded that the combination of levothyroxine and Sustanon has a synergistic effect in enhancing the neurogenic contractile response.

In this investigation, hypothyroid bladder strips showed reduced ATP-induced purinergic contractile responses in comparison to control bladder strips, which showed improvement with all treatment regimens. Hess et al^18^ observed the same contractile response to ATP in bladder strips from thyroidectomized rats compared to strips from control rats. Previous studies exhibited that hypothyroidism is linked to alterations in the purinergic system and increased ATP, adenosine monophosphate, and adenosine diphosphate hydrolysis in rat blood serum, hippocampal, and cortical slices.^19,20^ This finding suggests that purinergic contractile responses in the detrusor smooth muscle are likely to be lower in hypothyroidism caused by PTU and that changes to the purinergic system can be restored with any treatment plan.

The current study showed a decrease in KCl-induced contraction in the hypothyroid group compared to the control group and all treatment groups. However, the alteration was not statistically significant. A previous study showed that thyroidectomy did not alter the contractile responses to KCl in rat bladder strips.^18^ In another study, KCl-caused contractile responses in the rat urinary bladder were inhibited after thyroidectomy.^16^ These findings suggest that hypothyroidism may alter the intrinsic contractile state of the bladder muscle, depending on the experimental model and the duration of hypothyroidism.

In the present study, the smooth muscle-collagen ratio was reduced in the hypothyroid bladder tissue compared to controls. Monotherapies partially restored the reduction, whereas the combined therapy ultimately improved it. No previous data evaluates the effect of hypothyroidism on the smooth muscle-collagen ratio in the bladder. Furthermore, earlier data reported that castration reduced the ratio of smooth muscle to collagen and increased connective tissue in the urinary bladder, whereas TRT normalized the amount of smooth muscle.^9,21^ According to the results, an increased amount of smooth muscle is more linked to T levels than thyroid hormone levels; thus, treatment with levothyroxine may not guarantee the amendment of hypothyroidism-induced bladder dysfunction.

Urinary bladder function depends upon normal detrusor muscle function in the storage and voiding phases.^22^ Previous data imply that there may be a link between hypothyroidism and the severity of stress urinary incontinence and an opposite link between hypothyroidism and storage symptoms.^23^ Also, there was a decrease in micturition frequency and urine volume in patients with hypothyroidism.^2^ Furthermore, paralytic ileus (colonic pseudo-obstruction) was related to bladder atony in hypothyroidism.^5,6^ On the other hand, serum T level was reduced in men with hypothyroidism.^24^ The reduction in T levels is likely to have an impact on bladder function. Consequently, it is critical to diagnose and treat hypothyroidism while treating hypogonadism for optimal clinical outcomes.

There are a few limitations to the recent study. One of the limitations is that the levels of TSH were not measured to verify the PTU-induced hypothyroidism. It was shown that T3 and T4 levels in PTU-treated rats were lower than in control rats. Earlier data demonstrated a decrease in T3 and T4 levels and increased TSH levels in rat blood samples after 4 and 6 weeks of PTU administration.^13,25^ Thus, the administration of PTU is adequate to establish a hypothyroidism model via alterations in TSH and thyroid hormone levels. Furthermore, another limitation of the study is that bladder contractile responses obtained from organ bath experiments are not supported by functional assessment. No research has examined thyroid hormone and TRT for hypothyroidism-induced bladder dysfunction. Future urodynamic investigations in animal models or assessments of physiological responses to hormone replacement therapy under hypothyroid conditions should be conducted.

Finally, combination treatment with levothyroxine and Sustanon had a better impact on hypothyroidism-induced bladder dysfunction than monotherapy by improving bladder contraction and fibrosis. The results suggest that the combination of thyroid hormone and T may improve the treatment of hypothyroidism-induced bladder dysfunction with hypogonadism.

## Figures and Tables

**Table 1.** Characteristics of Animals

	Control Rats	Hypothyroid Rats	Hypothyroid Rats Treated with Levothyroxine	Hypothyroid Rats Treated with Sustanon	Hypothyroid Rats Treated with Levothyroxine + Sustanon
Body weight (g)	545.0 ± 15.0	332.8 ± 12.2***	353.8 ± 21.9***	366.6 ± 18.1***	368.8 ± 18.6**
Bladder weight (g)	0.24 ± 0.04	0.18 ± 0.02	0.19 ± 0.02	0.19 ± 0.01	0.20 ± 0.01
Total T (ng/mL)	6.71 ± 0.04	1.44 ± 0.09***	3.60 ± 0.57***	7.26 ± 0.25^###^	5.15 ± 0.5^###^
T4 (ng/dL)	0.89 ± 0.09	0.23 ± 0.01***	0.78 ± 0.01^###^	0.23 ± 0.01***	0.77 ± 0.01^###^
T3 (ng/dL)	0.168 ± 0.010	0.072 ± 0.003***	0.126 ± 0.001^##^	0.081 ± 0.02***	0.134 ± 0.016^###^

*P < .05, **P < .01, ***P < .001 vs. the control group.

^#^*P* < .05, ^##^*P* < .01, ^###^*P* < .001 vs. the hypothyroid group (ANOVA, Bonferroni post hoc).

## Data Availability

The data of this study is available upon request to the corresponding author.
